# Internet Protocol Television for Personalized Home-Based Health Information: Design-Based Research on a Diabetes Education System

**DOI:** 10.2196/resprot.3201

**Published:** 2014-03-10

**Authors:** Kathleen Mary Gray, Ken Clarke, Mabel Kwong, Basil Alzougool, Carolyn Hines, Gil Tidhar, Feodor Frukhtman

**Affiliations:** ^1^Health and Biomedical Informatics CentreUniversity of MelbourneParkvilleAustralia; ^2^Institute for a Broadband Enabled SocietyUniversity of MelbourneParkvilleAustralia; ^3^Diabetes Australia – Vic Proprietary LimitedMelbourneAustralia; ^4^LivBetter Group, formerly known as SeeCare, Proprietary LimitedMelbourneAustralia; ^5^Ericsson Proprietary LimitedMelbourneAustralia

**Keywords:** consumer health information, diabetes mellitus, health literacy, Internet, IPTV, patient education, social media, telemedicine, television, Web applications

## Abstract

**Background:**

The use of Internet protocol television (IPTV) as a channel for consumer health information is a relatively under-explored area of medical Internet research. IPTV may afford new opportunities for health care service providers to provide health information and for consumers, patients, and caretakers to access health information. The technologies of Web 2.0 add a new and even less explored dimension to IPTV’s potential.

**Objective:**

Our research explored an application of Web 2.0 integrated with IPTV for personalized home-based health information in diabetes education, particularly for people with diabetes who are not strong computer and Internet users, and thus may miss out on Web-based resources. We wanted to establish whether this system could enable diabetes educators to deliver personalized health information directly to people with diabetes in their homes; and whether this system could encourage people with diabetes who make little use of Web-based health information to build their health literacy via the interface of a home television screen and remote control.

**Methods:**

This project was undertaken as design-based research in two stages. Stage 1 comprised a feasibility study into the technical work required to integrate an existing Web 2.0 platform with an existing IPTV system, populated with content and implemented for user trials in a laboratory setting. Stage 2 comprised an evaluation of the system by consumers and providers of diabetes information.

**Results:**

The project succeeded in developing a Web 2.0 IPTV system for people with diabetes and low literacies and their diabetes educators. The performance of the system in the laboratory setting gave them the confidence to engage seriously in thinking about the actual and potential features and benefits of a more widely-implemented system. In their feedback they pointed out a range of critical usability and usefulness issues related to Web 2.0 affordances and learning fundamentals. They also described their experiences with the system in terms that bode well for its educational potential, and they suggested many constructive improvements to the system.

**Conclusions:**

The integration of Web 2.0 and IPTV merits further technical development, business modeling, and health services and health outcomes research, as a solution to extend the reach and scale of home-based health care.

## Introduction

Broadcast television has an almost universal presence and prevalent influence in homes. It remains the preferred mass information and communication medium among substantial numbers of people who do not use an Internet-connected device as their primary source of news and entertainment. Unfortunately for consumer health information and patient education, broadcast television does not provide health information that is reliably understandable and appropriate for any individual viewer.

Internet protocol television (IPTV) offers services not provided by broadcast television, for example, replays of television shows at viewer-selected times, interaction with live television shows, or video on-demand. IPTV is defined as “where television services are delivered using Internet Protocol over a managed broadband network”, and is thus distinct from simple Internet-connected TV, unmediated access to Internet content, or a hybrid of these [1]. IPTV has been described as “the killer application” of broadband Internet [2]. Although that prediction seems so far unfulfilled, it is in active use in many countries and it is the focus of ongoing international standards development [3]. A recent industry report identifies over 100 vendors and 940 service providers [4].

IPTV as a medium for consumer health information is a relatively underexplored area of medical Internet research. The idea that providing health information via television may improve engagement among people with literacy deficits has been around for some time: interactive TV among culturally diverse groups of patients and their health care providers was mentioned as an area for research in 2006 [5]. A recent systematic review identified 25 studies in the broad area of using IPTV and other interactive or digital television technologies in health, and noted the need for further evidence on which to base specification of user requirements [6]. Subsequent research papers have described designs for IPTV systems for consumer health information, for instance to provide medication reminders [7] and health videos [8]. A recent survey of potential providers and consumers of health information via IPTV offers in-principle support for such systems [9]. However, there is a gap in implementation and evaluation research.

The technologies of Web 2.0 add a new and less explored dimension to IPTV’s potential [10]. In general it has become technically possible to integrate Web 2.0 technologies with television to build systems that enable intelligently personalized recommendations and selections of television content [11,12]. The authors’ review of research literature has found just one example of a proposed design for health care services of this type [13].

Our research aimed to explore an application of IPTV for personalized home-based health information in diabetes education, in the context of a national high-speed broadband Internet infrastructure [14]. Type 2 diabetes is a major chronic health issue and health literacy is a factor in its prevention and management [15]. The Internet has created the challenge for diabetes educators to “push situation and user-specific quality knowledge to users based on their actual individual needs, circumstances and profiles at any given time” [16]. The Internet has also created the challenge for people with diabetes to become competent computer and Internet users or risk missing out on the flow of recent Web-based information and education.

Health literacy is “the degree to which individuals have the capacity to obtain, process, and understand basic health information and services needed to make appropriate health decisions” [17]. Better levels of health literacy can increase quality of life, optimize utilization of health care services, and reduce the burden of disease [18]. However, health literacy levels are surprisingly low [19,20] and there is a complex interdependency among different kinds of literacies that contribute to health and eHealth literacy [21,22]. To improve health literacy levels experts have recommended more personal forms of communication and educational outreach with “significant widening of the content and methods used” [23]; this approach has given rise to a wide range of information technology solutions for diabetes self-management [24].

We hypothesized that it is technically feasible to develop an integrated “IPTV 2.0” system. We theorized that such a system can enable diabetes educators to deliver personalized health information directly to people with diabetes in their homes; and that this system can encourage people with diabetes who make little use of Web-based health information to build their diabetes literacy via the interface of a home television screen and remote control. This paper aims to report empirical findings about the implementation and evaluation of an integrated IPTV 2.0 system.

## Methods

### Design-Based Research

**Figure 1:**
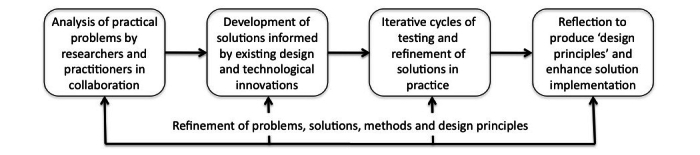
Design-based research process (attributed to David T Jones, sourced from Flickr and reproduced under a Creative Commons License).

It is not a simple matter for designers of Internet-based health interventions “to take a learner-centered, needs-based approach and to consider how all technology features (eg, text, graphics, interactivity, video, and games) can be used in ways to best meet the needs of learners” [25]. Because of the complexities of working ethically with an innovative Internet technology among people with diabetes and low literacies, this project took a design-based approach as illustrated in Figure 1.

Stage 1 of our research, corresponding to the analysis and development steps in Figure 1 and described further below, comprised a feasibility study into the technical work required to integrate an existing Web 2.0 platform with an existing IPTV system, populate it with content, and implement it for user trials in a laboratory setting. Stage 2 of our research, corresponding to the testing and reflection steps in Figure 1 and described further below, comprised an evaluation of the system by consumers and providers of diabetes information (ie, people with diabetes, their carers, and their diabetes educators). Our evaluation protocol was derived from a framework for measuring the International Standards Organization concepts of software product quality and system quality in use, based on capturing usability and user experience from different stakeholder perspectives [26]. Our participant numbers (4 providers and 13 consumers) met conventions for evaluation of usability [27]. Stage 2 proceeded with human research ethics approval from The University of Melbourne and Diabetes Australia-Vic (DA-Vic), and with the informed consent of all participants. The research was conducted from 2012 to 2013.

### Stage 1: Development of SeeCare Ericsson IPTV (S-IPTV) System at the Australian Broadband Applications Laboratory for Diabetes Australia (Vic)

The Web 2.0 service underlying this project was SeeCare [28], a service that enables consumers to manage their own home- and community-based care and support. Personalized health information content is provided based on people's specific needs, goals, and health conditions as these change over time. A consumer has their own SeeCare account and through this manages their own care, or they can give permission to a family member or an informal carer to act in this role if unable to do so for themselves. The Web tool provides a directory of care services and support agencies through which the consumer can arrange various forms of assistance with independent living at specified times. The care services and support agencies have Web-based access to only the level of information about the consumer that the latter chooses to make visible to each one.

Ericsson’s IPTV network infrastructure used in this project has been described elsewhere [29] as has the potential to develop a community education application as a partnership between Ericsson and SeeCare [30]. This partnership was fostered in the Australian Broadband Applications Laboratory (ABAL), a high-capacity broadband test-bed at the University of Melbourne’s Institute for a Broadband Enabled Society [31]. DA-Vic, the health service partner in this project, is a well-established not-for-profit peak body representing people affected by diabetes and those at risk [32]. The video content for the trial was supplied by Real Time Health, a company that specializes in providing digital video of patient narratives [33].

Video content in this project consisted of filmed interviews with people with diabetes and their carers, each interview relating their experiences with many aspects of this health condition such as symptoms, lifestyle choices, exercise, diet, mental health, and other issues. Each interview was segmented into several 5- to 10-minute videos, and each short video was identified with one or more health condition–related categories using keywords such as “depression” or “medication”. The videos were loaded onto the IPTV server and allocated an identification (ID) tag.

The SeeCare system can match a particular video’s ID tag automatically with a health care consumer’s needs, as specified by the health condition–related keywords they have in their account profile (this function was not trialed in this project). Alternatively a health educator logged into the SeeCare system at their workstation, and with permission to access a client’s S-IPTV account, can manually allocate appropriate videos to their client based on their expert opinion of the client’s information needs and wants (in this trial educators’ expert opinion was formed through a telephone conversation with each client).

After matching in SeeCare, ID tags and condition categories are then forwarded to the Ericsson server via the middleware, which acts as the intermediary in the transactions. This application programming interface specifies how the software components interact with each other to overcome any data format and exchange issues between the two separate systems. For privacy reasons the two systems share information only about the videos and health condition–related categories required for display, and not any other personal or medical information about the health care consumer.

**Figure 2:**
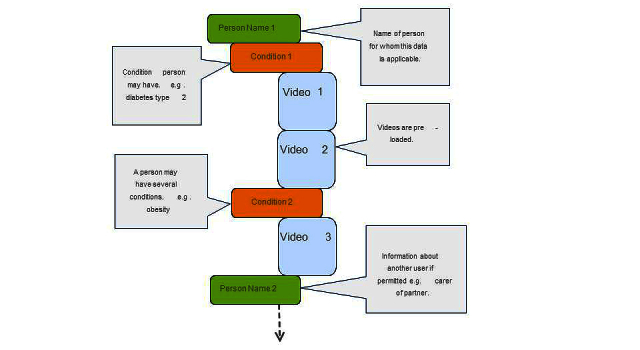
Dynamic user-specific data passed from SeeCare to Ericsson server.

**Figure 3:**
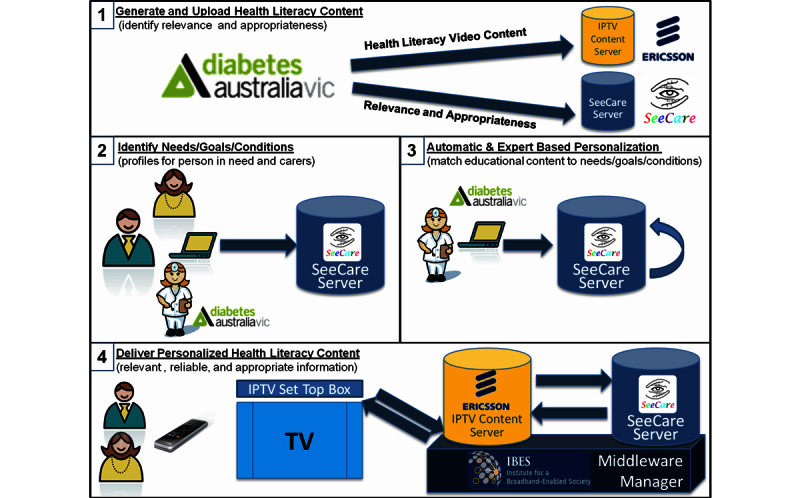
SeeCare IPTV workflow.

Through this process videos on distinct topics are made available, often more than one in each category, for a specific client to watch when they log in to their S-IPTV account. Figure 2 illustrates the data flow and Figure 3 illustrates the workflow.

The consumer interface consists of a large screen television connected to the Internet via a set top box, which decodes the incoming broadband data from the remote Ericsson IPTV video server and provides a high definition picture. The client must log in to the S-IPTV system via their television screen using a remote control before they can watch their assigned videos. This is not a technical limitation but part of the privacy requirements to ensure no one else can access a user’s account without authorization. The log-in process is as close as a consumer gets to requiring computer skills. In this trial, users had three different remote controls they could choose: a standard TV remote as well as two different types for the set top box. The first of these was a standard hand-held device with alpha-numeric buttons, and the second was a special keyboard-style remote to make typing of usernames and passwords easier for users who needed this.

After log-in the user can see a menu of health condition–related categories down the left side of the screen with thumbnails and text descriptions of each video title to the right. The remote control can be used to scroll down and across to the desired selection and the video can be played, paused, and fast forwarded as if the television were connected to a video player in the room, even though the content is being streamed over a broadband network from a distant server.

The system can handle multiple accounts and passwords so that family members and carers can access their own video selections; for example, there might be videos on meal preparation or wound management that carers specifically need to see.

### Stage 2A: Evaluation by Diabetes Educators

#### Participants

Four DA-Vic educators participated in the S-IPTV trial. Screening questions established their computer and Internet literacy. All educators used computers routinely for work and were competent at sending and receiving emails, working with standard word processors and spreadsheets, using an enterprise database, doing Web-based searches, and using mainstream social media platforms.

#### Measures

A researcher noted any initial barriers or enablers encountered by educators as they learned to use the S-IPTV system on a desktop computer at ABAL. Educators were asked to comment later about their experience with S-IPTV based on questions shown in the Textbox.

#### Procedures

A researcher set up a SeeCare user account for each educator. Educators attended a 1-hour long group presentation by researchers at ABAL, during which they had hands-on instruction on a personal computer about the basic functions of S-IPTV, including how to match video content from the system with the individual interests of a client. Each educator was allocated several participating DA-Vic clients, not previously known to them. From their workplace, using a script devised for this project, educators telephoned these clients to determine what each one would like to know about diabetes. While on the phone, the educator logged on to SeeCare on their desktop computer, selected video content suited to the client’s expressed information needs, and triggered its loading into the client’s S-IPTV account. After the client trials concluded, educators took part in a semistructured 1-hour group interview.

### Stage 2B: Evaluation by People With Diabetes and Their Caregivers

#### Participants

Participants were adult members of DA-Vic who had been diagnosed with Type 2 diabetes in the previous 12 months and had a low level of computer literacy. We chose to focus on computer literacy as a factor in health literacy based on evidence of a clear correlation between these two [34]. Initial recruitment of people with diabetes was done through advertisements posted in the DA-Vic main office and mailed to those on the DA-Vic mailing list. Volunteers phoned a researcher who used screening questions to exclude those with average or above average computer and Internet literacy.

Ten people with Type 2 diabetes participated, plus their spouse who acted as their informal caregiver in three cases, representing appropriate genders, ages, and fair to poor computer literacy levels. All participants used a television at home and some other technologies. Table 1 shows participant demographics and technology usage details.

**Table 1:** Participating people with diabetes and their caregivers (N=13).

Characteristics		n (%), N=13
**Gender**		
	Male	8 (62)
	Female	5 (38)
**Age**		
	65-80	9 (69)
	45-65	4 (31)
**Computer at home**		
	Yes	8 (62)
	No	5 (38)
**Internet connection at home**		
	Yes	5 (38)
	No	8 (62)
**Computer/Internet uses**		
	Word processor/spreadsheet	7 (54)
	Searching the Web and visiting websites	5 (38)
	Email	4 (31)
	Social media	4 (31)
**Frequency of computer use**		
	More than once a week but less than once a day	5 (38)
	No more than twice a month	8 (62)
**Other information and communication technologies at home**		
	Television	13 (100)
	Landline phone	13 (100)
	DVD player	12 (92)
	Mobile phone	5 (38)
	Gaming console	2 (15)

#### Measures

A researcher noted any initial barriers or enablers encountered by people with diabetes and their carers as they learned to use the S-IPTV system at ABAL. People were asked to comment later about their experience with S-IPTV based on questions shown in Textbox 1.

**SeeCare IPTV evaluation protocol.**

1. Health information providers and consumers.

- What videos did you watch on the S-IPTV system?
- How informative or uninformative did you find the S-IPTV videos?
- How relevant or irrelevant to your circumstances were the S-IPTV videos?
- What did you think about the variety and quality of the videos that the S-IPTV system made available to you?
- How would you describe the information for system users that the S-IPTV system made available to you?
- What reflections or related thoughts occurred to you while you were using the S-IPTV system?
- To what extent did you feel confident or unconfident about using the S-IPTV system?
- To what extent did you find it easy or difficult to use the S-IPTV system?
- To what extent did you find the S-IPTV system responsive or not responsive to your commands?
- To what extent would you say that the S-IPTV system was safe or risky to use?
- To what extent did you find the S-IPTV system enjoyable or problematic to use?
- To what extent did you like or dislike using the S-IPTV system?
- What was most interesting to you about the S-IPTV system?
- Would you want to use the S-IPTV system in future? Why or why not?
- What suggestions would you make for improving the S-IPTV system?

2. Health information providers only.

- For which people with diabetes did you select videos from the S-IPTV system?
- What was it like to use the S-IPTV system while on the phone with these people?
- What was it like to speak with these people about the S-IPTV system?
- To what extent do you think these people would find it easy or difficult to use the S-IPTV system?

3. Health information consumers only.

- To what extent would you say that you learned something from the S-IPTV videos?
- To what extent would you say that the S-IPTV videos inspired you to do something about your health?
- To what extent do you think your family, caregiver or doctor would use S-IPTV with you?

#### Procedures

Before the trial, each person with diabetes had an S-IPTV user account created for them and each person’s contact details were passed to a participating DA-Vic educator. They received a phone call from the educator inviting them to discuss their health condition, their knowledge of diabetes, and what diabetes information they would like to access on S-IPTV.

**Figure 4:**
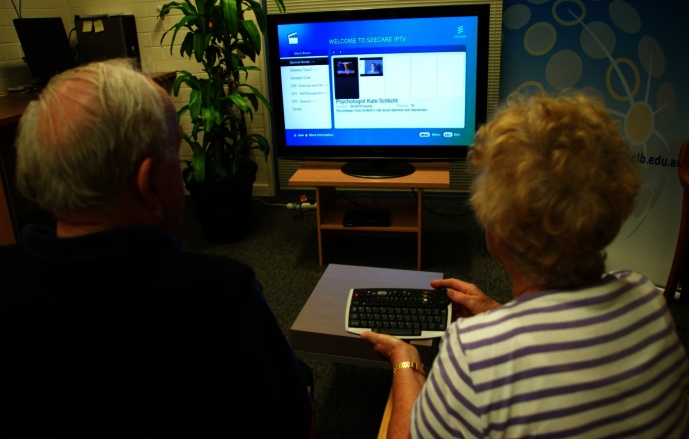
ABAL trial of SeeCare IPTV by a patient and a carer.

To trial the system, each person with diabetes (along with their carer in three cases) attended ABAL separately for a 1-hour private session with a researcher. There, to make the trial as natural as possible, they worked in a simulated domestic lounge or living room furnished with a television, easy chairs, and a coffee table as shown in Figure 4. Each participant was given a brief orientation to the project, including instructions and hands-on practice in using the basic functions of S-IPTV. Then they were invited to log in to their S-IPTV account, browse the menu of videos that their diabetes educator had created for them, and watch their choice of videos from this menu for 30 minutes. At the end of the session, they did a 20 minute semistructured interview about their experience.

### Stage 2 Data Analysis

One of the researchers transcribed audio recordings of ABAL user sessions, and analyzed these transcripts using open coding to arrive at initial themes and concepts. The researchers’ observational notes and video recordings of ABAL user sessions were reviewed to check and amend patterns in the interview data. Further thematic analysis was then conducted using selective coding; the initial themes were considered in relation to one another and regrouped in relation to the core concepts of usability and user experience. Coding was crossed-checked at several points by other researchers to ensure interrater reliability. Thematic analysis of consumer and provider groups was done separately, followed by comparison of findings from each group. Findings were arranged for presentation with regard to qualitative research reporting principles in health [35] and recommendations in eHealth [36], so as to provide rich description and interpretation rather than claims of statistical power.

## Results

### Key Findings

Key findings from the two groups’ evaluations of S-IPTV are reported for the major themes of usability and usefulness. Findings are categorized as subthemes within these, and also as enablers, barriers, or desiderata. Subthemes are illustrated with selected quotes.

### IPTV for Health Information Consumers

S-IPTV was found to enable health information access among people with diabetes and their carers through several usability and usefulness factors. Usability factors included familiarity with the television technology, simplicity of screen layout, and easy-to-launch video content. The major usefulness factors included a sense of affirmation, a sense of affinity, and a way to talk about the condition with family and friends. People with diabetes also identified usability barriers to health information access, namely the complexity of the remote controls, glitches in menus, and problems loading video content. They found usefulness barriers in terms of content that was unsettling, not relevant to their situation, or contrary to their idea of independently managing their condition. Their suggestions for improving the health information consumer experience of S-IPTV included work on manageability of remote control devices, screen readability, content metadata, richer types of content, follow-up features, and pathways to additional support. People with diabetes also made a number of suggestions about improving the S-IPTV log-in process and the graphical user interface, however researchers were not so concerned with this aspect of the pilot implementation as with more fundamental system quality observations. Examples of evaluation findings from this group can be found in Multimedia Appendix 1.

### IPTV for Health Information Providers

S-IPTV was found to enable health information provision by health educators through several usability and usefulness factors. Usability factors were fairly routine navigation and ease of selecting videos; usefulness factors were client convenience, wide appeal of videos, enhancement of educational methods, and alternative ways of performing their role as educators. Diabetes educators also identified usability barriers to health information provision, namely insufficient feedback from the system and excessive email exchanges needed to work with clients. They found usefulness barriers in terms of potential insensitivity toward clients, gaps in covering the breadth of topics required, the risk of appearing to offer a stand-alone self-education system, and the potential for system responsibilities to add to educators’ workload. Suggestions for improving the health information provider experience of S-IPTV included work on simplifying the educators’ user interface, providing simulations for educator training and development, developing client user support tools, expanding expert content, building in educational feedback, and person-to-person follow-up. Diabetes educators also made a number of comments about needing more opportunities to practice with the system before they would feel confident using it professionally; researchers recognize that this one-off trial for evaluation purposes was not equivalent to routine user education and training. Examples of evaluation findings from diabetes educators can be found in Multimedia Appendix 2.

## Discussion

### Strengths of IPTV

The project succeeded in developing a Web 2.0 IPTV system for people with diabetes and low literacies and their diabetes educators. The performance of the system in the laboratory setting gave them confidence to engage seriously in thinking about the actual and potential features and benefits of a fully implemented system. In their feedback they pointed out a range of critical usability and usefulness issues related to Web 2.0 affordances and foundations of good learning, not unexpected at this stage of system development, but valuable information from users’ perspectives. On balance, they described their experiences with the system in terms that bode well for its educational potential, and they suggested many constructive improvements to the system.

The outcomes of the technical feasibility stage of this project demonstrated that integration of an IPTV platform and a Web 2.0 platform can deliver functionality beyond that of “1.0 IPTV”. This functionality can be considered in terms of major strengths, weaknesses, opportunities, and threats, reflecting the researchers’ understanding of Web-based health information design and implementation issues.

The strength of S-IPTV is that it makes it possible not only to organize video content and optimize its use and reuse to meet the complementary needs of health information providers and consumers as groups but, further, to identify individuals in both groups and provide managed information services specific to each identity.

The weakness of S-IPTV is that it is not yet able to interoperate with other electronic health information management tools, such as patient records and patient monitoring devices, and has not been developed to meet health informatics standards, for example, in terms of metadata, access controls, or secure messaging, but, as an IP-enabled platform, it could be made to do so given sufficient time and effort.

The opportunity that S-IPTV affords is to develop business models that use a home television as a mode of communicating person-specific, professionally-selected health information, and thus to extend the reach and scale of home-based health services.

The threat that S-IPTV faces is that it may be superseded by an IPTV platform, which adds more Web 2.0 interactivity to the provision and consumption of health information, for instance user-generated content and shared feedback on content as seen in peer-to-peer health social networks.

Some of these considerations are echoed clearly in the findings from user evaluation of the project. For example, there are usability comments about the user-friendliness of the technology, the need to streamline email exchanges between educator and client, and improvements to the composition and description of videos. There are usefulness comments about the value of a close fit between the content and the viewer’s personal situation, the need to think of this system in relation to other information systems, and the desirability of adding extensibility and interpersonal interaction functions.

Apart from personalization, some other characteristics associated with Web 2.0 interactivity (manipulating and enhancing multimedia content, sharing narratives, building social networks) also emerge from users’ suggestions as possible directions for further convergence between IPTV and Web 2.0.

The findings from the evaluation stage of the project show considerable alignment in clients’ and educators’ perceptions about the potential for S-IPTV to enable health literacy. Both groups described or could envision user experiences that highlight important elements of learning, such as learner-centeredness, affective responses, contextualized content, time to reflect, timely feedback and follow-up, and making learning social.

Some clients’ and educators’ comments also flag nontrivial barriers to using S-IPTV for health literacy. There are consumer perceptions that the video content about diabetes may be uninformative or even distressing, and that watching videos is not an effective part of responsibility for health self-management. There are provider perceptions that the system needs additional elements to ensure safety and quality in the provision of information, and that the service needs to be more closely integrated with a range of complementary information and education services.

### Limitations

No attempt was made to look for statistical inferences, given that data from a pilot study of this size are not likely to support quantitative analysis. This was the key limitation of this study. A larger sample size most likely would have yielded more significant results about the usability and usefulness of the system. Future research with a larger sample size of health information consumers would make it feasible to compare the perspectives of those with varying levels of literacies, and other demographic factors. Future research with a larger sample size of health information providers would similarly support quantitative findings about usability and usefulness in terms of professional work practices and quality of health care services. Nevertheless, this study was appropriate to evaluate an initial version of the S-IPTV platform with a group of computer illiterate consumers and a group of busy professional providers, and it has made an original contribution to expanding knowledge not only about the design of IPTV 2.0 for health care, but also about its implementation in a realistic setting.

### Conclusions

To our knowledge, this was the first study of its kind to address consumer health information needs by building a working IPTV system that integrates Web 2.0 technologies, conducting an implementation trial with health information providers and their clients, and evaluating the user experience. This project achieved its aims. It succeeded in integrating a Web 2.0 approach to content personalization with an IPTV system, turning the television and remote control into much more than simply a different user interface to the Web. It demonstrated that such a system was seen to be usable and held much interest, among people with high self-management needs and low literacies and among their health educators. It yielded insights into the user experience of a Web 2.0 IPTV system for health information, from both consumer and provider perspectives.

Additional stepwise improvements taking account of these insights is consistent with a holistic view of process and outcome in successful eHealth innovation [37]. Further technical development of S-IPTV is needed to improve some of the current communication and interface issues, and to take greater advantage of opportunities for interactivity and interoperability. Further development of S-IPTV as an information and education system is needed to formulate a model for content production and use that is more sophisticated, and a model for service delivery that is more strongly integrated with other home-based care and support programs. Further research into S-IPTV is needed to study its use by a wider range of health information consumers and providers, in more naturalistic settings, particularly to understand the impact it may have on health care services and health outcomes.

This research has contributed empirical research evidence that IPTV influenced by Web 2.0 has the potential to transform the apparently old technology of the home television into an innovative and wide-reaching tool to improve health literacy, especially among some under-served groups. The implications of low health literacy for population health and for the sustainability of health care services are so great that IPTV merits substantially more attention than it has received so far.

## Multimedia Appendix 1

Evaluation by people with diabetes (caregivers [C] are identified in relation to the person [P] they accompanied, eg, C8 was the caregiver who accompanied P8).

## Multimedia Appendix 2

SeeCare IPTV evaluation by diabetes educators.

## Abbreviations

ABAL: Australian Broadband Applications Laboratory
DA-VIC: Diabetes Australia–Vic
ID: identification
IPTV: Internet protocol television
S-IPTV: SeeCare Ericsson IPTV
