# Metadata Correction: Internet Protocol Television for Personalized Home-Based Health Information: Design-Based Research on a Diabetes Education System

**DOI:** 10.2196/resprot.3404

**Published:** 2014-03-18

**Authors:** Kathleen Mary Gray, Ken Clarke, Mabel Kwong, Basil Alzougool, Carolyn Hines, Gil Tidhar, Feodor Frukhtman

**Affiliations:** ^1^ Health and Biomedical Informatics Centre University of Melbourne Parkville Australia; ^2^ Institute for a Broadband Enabled Society University of Melbourne Parkville Australia; ^3^ Diabetes Australia – Vic Proprietary Limited Melbourne Australia; ^4^ LivBetter Group, formerly known as SeeCare, Proprietary Limited Melbourne Australia; ^5^ Ericsson Proprietary Limited Melbourne Australia

The author Mabel Kwong was inadvertently omitted from the list of authors during the production stage of the paper "Internet Protocol Television for Personalized Home-Based Health Information: Design-Based Research on a Diabetes Education System" (JMIR Res Protoc 2014;3(1):e13). The author Mabel Kwong (Institute for a Broadband Enabled Society, University of Melbourne, Parkville, Australia) should have been added after Ken Clarke in the original published manuscript. This error has been corrected in the online version of the paper on the JMIR Research Protocols website on March 18, 2014, together with publishing this correction notice. This was done before submission to Pubmed Central and other full-text repositories.

