# Supplementary material for: Internet Protocol Television for Personalized Home-Based Health Information: Design-Based Research on a Diabetes Education System
Source: JMIR Res Protoc. 2014 Mar 10;3(1):e13. doi: 10.2196/resprot.3201 (PMC3967124; doi:10.2196/resprot.3201)
Supplement: Supplementary file 1 [file resprot_v3i1e13_app1.pdf]

Theme	Health information enablers	Health information barriers	Suggestions
**Usability**			
Devices	Familiar technologies:<br>P1: It’s quite easy to use, not much of a problem, it’s just learning the commands […] and the TV. That’s it. I don’t have any problems with that.<br>P4: Now the fact that I’m familiar with this is because I got exactly the same. I got the same TV, so there’s nothing new.<br>P5: It’s simple to use. It’s not complicated to use. As long as you know how to use your set-top box controller [gestures to set-top box remote] and you’re generally familiar with how Internet connected television sets work, or you use computers, you should be able to work out how to use it fairly simply.<br>P6: It’s no different from my pay-TV controller or DVD controller.	Configuration of remotes:<br>P1: It would be good if you could have all of them on one remote.<br>P4: If I ever wanted to change something or some channel […] that to me is a lot troublesome, because you’re fixing one and you [mess up] the other ones. So I don’t want to touch [it].<br>P5: That’s the smallest keyboard I’ve ever seen in my entire life and I wouldn’t have a keyboard that small in my house. I would suggest a bigger one.<br>P9: If you’re working with electronic things like computers you’d probably work with the remotes a lot quicker than I did because our remote [at home] is working the television set, going backwards and forwards from ordinary television to [pay-TV] or something.	Manipulability:<br>C8: There’s a lot of playing around with them […] It would be handy to have one, I suppose [makes pressing remote motion with hands].<br>C9: I probably have difficulty looking at the numbers and the things on them […] they could be bigger [gestures at the buttons on the top right of the keyboard].
On-screen navigation	Simple to decipher:<br>P3: You can see the words clearly and it’s not too big or it’s not too bright. I’d say it’s about the right size.	Menu detail:<br>P4: You got rating 10. I don’t understand what it means by your rating 10.	Readability:<br>P5: The font wasn’t great and it could be better. To be honest, it kept shifting from

	P5: Generally it's a nice screen and I think it's big enough.	P5: [I'm assuming] all the information will be Type 2 diabetes related, so I don't think you need to tell me each topic is Type 2 diabetes […] it's overkill to have it on every single menu item.<br>P7: When you were watching the headings there, it's not clear exactly what the heading is (menu bar cut-off).	top to bottom. You make up your mind. I'm either [moves hands up and down]. And it could be a bit better in terms of type of font.
Choosing to view	Easy to play:<br>P1: I like the subtitles, that was very good […] I could read them clearly.<br>P6: I could jump around and pick the order that I wanted to do things in.<br>P8: When I pressed the button it got up there. It was good.<br>P9: Soon as I knew what I was doing, I was confident. I don't have any worries with that.	Hard to play:<br>P5: When I pressed buttons, it was slow to move… they're [videos] slow at loading.<br>P6: Frustrating in that you get halfway through a sentence and or most of it, and chopped off.	Video metadata:<br>P1: Specific titles so people with specific problems can go and look up their specific situation.<br>P5: The duration […] could be more user-friendly in relation to just saying 5 minutes, not 0000005 hours.<br>P5: There's nothing in there [gesturing to a video's description] that tells me what she's going to tell me about... It needs to be a bit more, what am I going to learn when I watch this video.
**Usefulness**			

Affect	Affirmation:<br>C2: It's reassuring to see people that talk about this disease.<br>P6: When you go through a bad period – because everybody has a bad period – it's nice to get on, hear and find other people who had exactly the same thing and how they coped.<br>P9: It will obviously go out to people who don't know enough [about diabetes] and they'll listen to other people and think 'Oh. Yeah.'<br>C9: For someone who's just been diagnosed with diabetes, you're not 'mmmm' [sags body and shoulders]. It gives them hope.<br>P10: It's comforting […] Because the worst mightn't happen to you.	Unsettling content:<br>P3: People getting their toes chopped off and that sort of stuff, you'd have to be very careful how it was handled, you wouldn't want to frighten people or put people off having treatment. It's probably a fairly delicate area.<br>P5: That's extreme [Name of support service], an extreme link. That's suicide prevention. I would've thought that seems way over there somewhere.<br>P6: Some of that might scare you. Knowing that you can have tingly feet and the rest of it is [not] a nice thing to look forward to.<br>C8: That will scare them into doing [self-management], what can happen to your body.	Enrichment:<br>C2: Maybe putting extra people in there to give more opinions […] More people, any age group.<br>P4: I would've liked to see more on the side of research […] there's always hoping to get one day when you can get rid of it.<br>P5: You might give the anecdote and then you might have the facts up alongside the anecdote.<br>C9: Instead of just talking there, show a few exercises that would help you.
Personalization	Affinity:<br>P3: The exercising and dieting one was more interesting to me.<br>P6: It's nice to hear that other people have the same symptoms as you had and the same problems you've had.<br>P7: It was also interesting to see how you can depend on your family for support. In my case, my wife	Irrelevance:<br>P1: People describing their condition with diabetes and their experiences […] Everyone seems to be suffering from depression. Which I don't seem to suffer from […]. I want to watch someone towards, around my age.	Follow-up:<br>P5: What would be brilliant is if you could print something off. Like if there was a paper or a link to a website that you could actually go from here to the website and download a piece of material that

	suffers from depression as well, so we support each other in that respect.<br>P8: I can relate to nearly all of them […] This lady here, I could relate to her because I haven't been well for two or three weeks and I was working at that time.<br>P9: Only one of them said that they were very thirsty. That was in my case.	P4: It's what I already know.<br>P5: It's just anecdotal. It's interesting to hear what other people are experiencing […] but do I get value from it? No. Do I learn anything? No.	supports what you just learnt.<br>P6: You use it, you go through and watch the ones you want to watch. And then in a week or month's time, you pop back again to see if any new ones have cropped up, which could also be reinforced by emails saying "new content is available".<br>P9: That mightn't be a bad idea on there, to have a little instruction that can be printed out […], a how-to guide.
Connectedness	Friends and family:<br>P4: I have friends of mine that became diabetic recently […] I'm trying to explain to them what's in it and what you're encountering [...] I would show it to them, I would say, 'that's what you've got to do, listen to this, watch this and try to learn'.<br>P5: I'd probably use it also to help teach my children the signs and stuff.<br>P7: I could show it to my sisters. These sisters are not very familiar with computers and stuff like that. Maybe	Personal responsibility:<br>P1: Self-management and having an understanding of your situation, that's the main thing.<br>C2: Once you get the hang of it, you'll probably get sick of watching it, 'I'd better stick to my plan'.<br>P5: I work full time. What time left in the day I have, I'd rather exercise than spend it watching a video.<br>P7: You suffer as the one who has got to take charge of your health […] especially the children just don't	Pathways to support:<br>P1: Maybe connections with organizations that exist, like exercise places and fitness places and places where people can go to.<br>C2: If you add an extra video to say, 'this is what you need to do with diabetes, it's very important to attend groups'.<br>P6: It would be nice to have more content from Diabetes Australia promoting the

	one day when [they] were home visiting me, I’d sit next to them, ‘I’ll show you this video’.	have the time [for IPTV].<br>C8: You become your own doctor. / P8: Yeah, you do become your own doctor in the end.<br>P9: We’ve been pretty thorough since I’ve got diabetes, going to diabetes educators, diabetes dietician. We went to courses on diabetes […] so we’ve learnt a lot about it. I don’t know if I learnt anything more from [IPTV].	activities that they have […] the programs they have.<br>C9: A list of things where you can get help, like the podiatrist and the physio, […] dietician, healthcare nurse […] just to give an idea of what can be done to help.
