# Supplementary material for: Internet Protocol Television for Personalized Home-Based Health Information: Design-Based Research on a Diabetes Education System
Source: JMIR Res Protoc. 2014 Mar 10;3(1):e13. doi: 10.2196/resprot.3201 (PMC3967124; doi:10.2196/resprot.3201)
Supplement: Supplementary file 2 [file resprot_v3i1e13_app2.pdf]

Theme	Health information enablers	Health information barriers	Suggestions
**Usability**			
Navigation	Routine:<br>E1: A lot of nurses, diabetes educators already use certain databases or whatever for their particular work and this is just another one, another platform they need to use.<br>E2: It responded to everything I asked it to do.<br>E3: Finding the videos, that was fine.<br>E4: It was easy to find my way around the site and to find the videos.	System non-feedback:<br>E1: I'm not clear still whether I did it properly...I don't know. Because I've not seen the other side. So I don't know has it come through.<br>E4: Maybe I used the system wrongly, I'm not sure.	Simplification:<br>E1: There were multiple ways to get into an area, and I think most of us worked out many ways, remembered different ways. I don't know it that's better or worse. Like whether just one way, a simpler way to get in.<br>E3: A bit convoluted. It could be simplified.
Content-client matching process	Ease of selection<br>E1: It was quick. Once you had the videos there, loaded up, to be able to click on and say, yes, this is what I want them to know, it's relatively quick. And apparently it's updated automatically<br>E2: Once you're in there it's very easy just to link videos.<br>E3: Personalizing videos is easy.	Email exchanges<br>E1: I got four emails saying this participant is there. Then I got another email saying that he's accepted me. […] And now it's been upgraded to this. Now it's been upgraded to that. That's a lot of emails when you get a hundred emails a day.<br>E3: I had to wait for an email to come to me and then I had to send the email back after the invitation to see they'd agreed. You assume that if they [clients] sent you the email, it implies agreement anyway and acceptance and I	Simulation<br>E1: [The system] probably need[s] a made-up patient or something or client so that you could put in information and practice.

		should be able to log on with their details and that information should be readily available once they've given that permission.	
**Usefulness**			
Client-centeredness	Convenience:<br>E3: They'll [clients] probably find it fairly easy. Most people know how to change stations and use the remote control. People probably these days get [Name of pay-TV service] so again they're probably fairly good at using that sort of technology.<br>E4: It's excellent for people [clients] to go in their own time […] and it does meet a need for people perhaps who don't feel comfortable with using the Internet, might be more comfortable with using the TV.	Insensitivity:<br>E1: There may be aspects of diabetes that can be very, very sensitive. Would I, as a patient, trust that? Probably not. If I wanted to discuss something personal, knowing that someone's fiddling around [in the system …] Could the information go somewhere else?<br>E3: If you do have any personal information and you don't want people being able to hack into it, to access it […] they [clients] don't want anyone else to see.<br>E4: Just being told to watch a video is a bit impersonal.	Client user support:<br>E4: A good explanation and perhaps a very practical demonstration of how to use it would be really important.<br>E4: In the future, is there going to be the option of providing some sort of further support rather than just the videos.
Subject matter	General appeal:<br>E3: Some of the personal stories that people told […] make it easier for people [clients] to relate to rather than listening to a health professional all the time, who they might be thinking 'doesn't understand what I'm going through	Selective coverage:<br>E1: They were more about the emotional aspects of diabetes, those videos. But there are also the more task-oriented […] those videos were the tip of the iceberg.<br>E3: Some things are targeted but you're	Expert content:<br>E3: Sometimes they [clients] may want a little bit more information or a little bit more detailed information or how to go about finding it.<br>E4: You might find that doing

	or doesn't know' […] having the personal stories can help that way.<br>E3: Even people who've got good literacy levels and things like that would still find something like that really good.	not targeting some of the other things.	some extra videos of diabetes educators or dieticians having a presentation might be helpful as well to answer some of the further issues that might not have been mentioned in detail.
Mode of education	Enhanced learning:<br>E1: It's more to enhance and for them [clients] to be able to say something and ask more questions if they learnt more about it.<br>E2: They [clients] can get a bit more information than the doctor or the diabetes educator can give them in one consultation.<br>E3: It gives them [clients] the time to have a look at the information for themselves at their own pace and they can reflect on it or go back and rewatch it, or if they didn't understand anything it just might also introduce other questions as well.<br>E4: It's a great way perhaps for people in the family, extended family, to have the opportunity to watch […] that mightn't get an individual appointment.	Not stand-alone:<br>E1: There are so many issues [cites the case of a specific client], I don't know if videos would be the only medium to support him [client].<br>E2: It's not enough for them [clients] to take that as gospel and that's the only thing they need for their management of diabetes.<br>E3: It can be an overwhelming amount of information.	Extensibility:<br>E1: You would give them [clients] the information you want plus you would use that as adjunct therapy, you would [say] 'I'm going to send you this and maybe contact me back for a review'.<br>E3: That would be good to be able to have some way that they [clients] can either email questions at the time that they're sort of thinking about it. Or some way of communicating any question so that they can be answered either immediately or at some other time.
Role of educators	Alternative way of working:	Review responsibilities:	Interpersonal interaction:

	E1: If you used it as part of your workload and you found that it reduced your workload, then you would say, ‘Wow. I can get a lot more done. I don’t have to post information [through the mail]. I can do it through this.’<br>E2: I would find it a good backup.<br>E3: The ability to remotely provide some information. There’s a place for that.	E1: If you were using other databases, that’s too much information […] If you had many systems, all those patient notes, and all that, I don’t know how much of that you need.<br>E1: We would have to review every video. / E2: And need to update it. / E1: Because I would want to know exactly what was on that video before I send things off.	E1: I needed to actually ask that question, ‘Do you need extra support, do you feel like you need to have a chat outside this little conversation we’re having now?’<br>E2: Something to say […] ‘should you need further clarification or assistance, you should seek your diabetes educator or health professional’.<br>E3: You still need one-on-one communication as well.<br>E4: There needs to be some interaction with a health professional as well.
